# Cerebrovascular reactivity: a stress test of brain vascular health in moyamoya disease

**DOI:** 10.1093/braincomms/fcae419

**Published:** 2025-01-11

**Authors:** Audrey P Fan, Meher R Juttukonda

**Affiliations:** Department of Neurology, University of California, Davis, CA 95817, USA; Department of Biomedical Engineering, University of California, Davis, CA 95616, USA; Athinoula A. Martinos Center for Biomedical Imaging, Massachusetts General Hospital, Charlestown, MA 02129, USA; Department of Radiology, Harvard Medical School, Boston, MA 02114, USA

## Abstract

This scientific commentary refers to ‘Cerebrovascular reactivity and response times describe recent ischemic symptomatology in patients with moyamoya’, by Han *et al*.  (https://doi.org/10.1093/braincomms/fcae381).

**This scientific commentary refers to ‘Cerebrovascular reactivity and response times describe recent ischemic symptomatology in patients with moyamoya’, by Han *et al*.  (** https://doi.org/10.1093/braincomms/fcae381 **).**

Prediction of stroke symptoms is a critical clinical need for patients with moyamoya disease. Moyamoya disease is an idiopathic cerebrovascular disorder that leads to progressive narrowing of major intracranial arteries that supply blood to the brain. The prevalence of moyamoya disease has increased in the past two decades, including in East Asian countries (where moyamoya disease is most prevalent) and in North America,^1,2^ in part due to wider use of non-invasive diagnostic imaging. The current imaging guidelines^1^ focus on structural imaging of affected brain vessels using digital subtraction angiography and MRI angiography to measure steno-occlusion as the primary diagnostic criteria. Similarly, the largest moyamoya disease registries such as the Japanese AMORE (Asymptomatic Moyamoya Registry) multi-centre cohort have focused on angiographic staging and structural lesions to predict future stroke occurrence in a large cohort of patients with moyamoya disease.^2^

However, such structural imaging provides limited information about the health of the brain tissue itself. This is true especially in earlier stages of ischaemic events before the onset of visible lesions—and when it is still possible to intervene to prevent symptoms. For patients with moyamoya disease, understanding tissue-level haemodynamic changes is even more pertinent because these individuals develop complex and heterogeneous collateral artery patterns, making it difficult to directly associate large-vessel changes with brain tissue health. Direct imaging of cerebrovascular function and properties of the brain is thus an important need. Establishing its benefit can inform future randomized control trials of revascularization surgery for moyamoya disease to prevent ischaemic stroke; there have been no such prospective trials despite routine use of direct and indirect bypass surgeries to treat stroke symptoms.

In their recent article in *Brain Communications*, Han *et al*.^3^ studied cerebrovascular reactivity (CVR) in a moderately large cohort of 73 individuals with moyamoya disease or syndrome using blood oxygenation level-dependent (BOLD) functional MRI. CVR is the perfusion response of brain tissues to an external stimulus, in this case imaged by BOLD while having participants breathe short periods of hypercapnic gas with slightly higher levels of 5% CO_2_. This breathing task serves as a ‘stress test’ for the brain, allowing the study to directly probe vascular function of cerebral tissues not only at rest but during a challenged condition. In directly monitoring CVR, Han *et al*. showed lower CVR in brain hemispheres associated with ischaemic symptoms within the past 6 months compared with asymptomatic hemispheres across the cohort. The predictive value of CVR was further reinforced by receiver operating characteristic curve analysis, enabled by the relatively large sample size of patients with moyamoya disease.

A major strength of the methods employed by Han *et al*. is leveraging the temporal resolution of the BOLD fMRI signal (sampling every 2 s) to measure the timing of the CVR response to the gas breathing task. The study further showed that the time to achieve the maximum perfusion response (CVR_delay_) was longer in symptomatic compared with asymptomatic hemispheres of patients, but with no difference in the amplitude of the response. CVR_delay_ was also highly predictive of ischaemic symptoms, outperforming ‘raw’ CVR measures in receiver operating characteristic analysis. This information provides mechanistic insight that changes in ‘overall’ CVR that have been observed in other studies may be driven in large part by altered timing of vascular responses in moyamoya disease (relative to the imaging). Han *et al*. provide strong evidence that CVR_delay_ is an important haemodynamic parameter and that it may ultimately be used to evaluate efficacy of revascularization therapies in clinical trials.^4^

In addition to identifying impaired CVR measures in the symptomatic hemispheres, the authors also presented how the maximal response (CVR_max_) and CVR_delay_ are associated with age in asymptomatic hemispheres of patients with unilateral disease. Overall, the inverse relationship between age and CVR_delay_ was found to be stronger than the association between age and CVR_max_. These results could then serve as normative data against which the affected hemispheres can be compared. Indeed, by plotting the corresponding value from the symptomatic hemisphere on these age curves, the authors evaluated the degree of impairment while accounting for age and using normative data from within the same patient. The efficacy of this approach is a testament to sample size of the current study and will only improve as more data are available. The investigators’ success in having dozens of participants breathe hypercapnic gas is also consistent with high success rate of gas-based CVR assessment 90.3% reported by other sites^5^ and portends well for broader use of CVR metrics in future studies of moyamoya disease and other cerebrovascular disorders.

In this study, CVR was measured by coupling the administration of a hypercapnic respiratory challenge with a BOLD MRI. This combination was chosen over other common stimuli, such as acetazolamide, and imaging techniques, such as gold-standard PET, given the resulting advantage of gleaning information about the temporal dynamics of the CVR signal in addition to the magnitude of the CVR response.

A block paradigm was used where two blocks (180 s) of hypercapnia with carbogen gas (i.e. 5% CO_2_/95% O_2_) were interleaved with a similar duration of medical-grade room air. The choice of carbogen gas as the respiratory stimulus carries the advantage of being more easily attainable for human use. However, as Han *et al*. have discussed, there is some evidence that carbogen is not entirely iso-metabolic and may result in a BOLD response that is independent of CVR. Given this, CVR measures obtained using carbogen may not be directly comparable to those obtained using standard CO_2_ (e.g. 5% CO_2_ with balance room air) or pharmaceutical approaches.

The respiratory stimulus was administered through a non-rebreathing face mask, and end-tidal CO_2_ was measured using MR-compatible equipment and a nasal cannula for sampling. Alternative approaches using computerized administration of the stimulus have also been introduced that allow for more precise control of blood gases, and these approaches may help mitigate potential inter-patient differences in respiratory physiology unrelated to vascular disease in future studies.^6^

A T_2_*-weighted MRI sequence tuned to be sensitive to the BOLD response was chosen as the readout approach used to acquire the CVR-weighted imaging data. As changes in blood flow and blood volume are the fundamental quantities of interest, arterial spin labelling MRI would be the ideal implementation for measuring the microvascular response to vasoactive stimuli. Such approaches have been used in the context of assessing CVR responses to pharmaceutical challenges.^7^ However, arterial spin labelling is slower than BOLD, requiring a pair of images to produce the required labelled contrast at a single time-point, which can take approximately four times longer than a single BOLD time-point. In addition, arterial transit times are known to be delayed in patients with moyamoya disease,^8^ and the choice of post-labelling delay could serve as an additional confound.

Finally, while there are advantages compared with pharmaceutical approaches, the administration of a respiratory challenge is also not trivial. Therefore, approaches such as breath holding that can introduce hypercapnia without external stimuli^9^ or that can utilize resting-state data to provide measures of CVR^10^ may be ideal for clinical translation in settings where respiratory challenge set-up may not be feasible. Han *et al*. have provided compelling motivation for CVR and related metrics to address an unmet need for routine, direct imaging of tissue-level brain vascular health and viability in moyamoya disease. The gas-based approaches adopted by Han *et al*. and related CVR imaging methods have the future potential to transform clinical studies and disease management of moyamoya disease.
